# Addressing biomedical complexity using the energy systems language

**DOI:** 10.3389/fsysb.2026.1903662

**Published:** 2026-10-07

**Authors:** Federico Surra, Francesco Gonella, Luigi Conte, Alessandra Romano

**Affiliations:** 1 DMSN-Department of Molecular Sciences and Nanosystems, Ca’ Foscari University of Venice, Venice, Italy; 2 SMAC-Centre for the Study of the Systemic Dynamics of Complex Diseases, Ca’ Foscari University of Venice, Venice, Italy; 3 NICHE-Centre for Environmental Humanities, Ca’ Foscari University of Venice, Venice, Italy; 4 DAIS-Department of Environmental Sciences, Informatics and Statistics, Ca’ Foscari University of Venice, Venice, Italy; 5 Department of General Surgery and Medical‐Surgical Specialties, University of Catania, Catania, Italy

**Keywords:** energy systems language, immune system model, quantitative biological models, systems biology, systems thinking

## Abstract

Biological complex systems self-organize and evolve through feedback-driven interactions not adequately captured by linear cause-effect models. To address this gap, we propose a novel approach grounded in Energy Systems Language (ESL). It provides a description of how resource availability, carrying capacity and feedback networks shape the system trajectories. ESL is rooted in open systems Thermodynamics, shifting the focus from the biology of events to their systemic structure. Models are built on biomedical knowledge, with parameters determined by empirical observation and biophysical constraints, producing simulators that address “what if” exploratory questions. We applied this approach to the antigen-specific adaptive immune response as a proof-of-concept of the framework designed to investigate system-level dynamic regime stability and qualitative response to external driving forces. We developed a computational simulator reproducing hallmark features of humoral immunity at daily, monthly and decadal time-scales, including germinal-center dynamics, establishment of long-lived plasma cells, antibody secretion, and long-term decline in the absence of antigen re-exposure. Using this approach, future investigations of complex pathologies, e.g., lymphoid malignancies and autoimmune diseases, can be developed, providing computational tools for testing hypotheses on patient-specific therapeutic strategies.

## Introduction

Certain classes of incurable chronic diseases, such as autoimmune disorders and some forms of cancer, exhibit complex behaviors for which comprehensive biological and medical descriptions remain limited. As a matter of fact, it is very difficult to frame characteristics such as chronicity, resurgence, plasticity, non-linearity, patient-dependence within clear cause-and-effect chains even from the disease onset. In fact, these are complex behaviors that stem from the deeply systemic nature of these features. This biological complexity—marked by a distinct dynamic character—emerges after the homeostatic functioning of a system is disrupted by external driving forces. In response, the system adapts by altering its configurational patterns. These adjustments modify systemic temporal behaviors, giving rise to the range of features that characterize the progression of pathological conditions across different time scales. It is therefore necessary to explore how systemic analytical tools used to describe complex dynamics may be used in medical research.

In general, as described for example in recent work by Gentili (Gentili, 2026), complexity can be approached by several well-assessed methods, notably those relying on computational tools and methods, including Network Analysis, Big Data and even Artificial Intelligence tools. But while all the approaches aim at describing a system as a whole, many of them still retain the scale of the microlevel, inasmuch as they consider a system as a collection of a large number of local elements (for example, nodes and links), whose dynamics is investigated thanks to the availability of big data and sophisticated mathematical and statistical tools. On the other hand, the ESL approach starts from the identification of a limited number of state variables, those necessary to model the flows of matter, energy and information, together with the feedback networks that describe the configuration patterns of the system dynamics. The capability of answering “what-if” questions is a fundamental feature needed for an effective modelling aimed at a dynamic description of a complex system. In this perspective, ESL is expected to provide the needed potential.

What we refer to as real flows are physical connections between stocks, or between stocks and processes. In a biological system—with the possible exception of units of space, which represent a kind of virtual energy—they are physically made of matter. Since both matter and information can be attributed an energetic content, ESL refers to flows of matter, energy or information as real flows, to distinguish them from the connections used, for example, in Causal Loop Diagrams or in some forms of Network Analysis. Control flows are a distinct case: they do not enter the outflow balance of a stock, because the energy involved in their regulatory action on a process is negligible with respect to the other mechanisms that deplete the stock.

Systemic approaches have been extensively proposed and employed in biology and medicine over the past decades (Hood et al., 2012), driven by the success of Network Analysis methods and the growing availability of large datasets. Concepts such as Systems Biology (Loscalzo and Barabási, 2011; Klipp et al., 2016) and Network Medicine (Kreeger and Lauffenburger, 2010; Barabási et al., 2011) are now widely applied, particularly in cancer research, providing powerful tools to characterize the organization and connectivity of biomedical systems. These approaches have been especially effective in revealing statistical, structural, and topological properties of complex biological networks, while the representation of system-level dynamics and the exploration of “what-if” scenarios are typically addressed through different modelling strategies. Indeed, frameworks that support the exploration of “what-if” scenarios and the analysis of leverage points are essential for investigating systemic responses to drug administration and therapeutic interventions (Romano et al., 2021; Cazzagon et al., 2021). This can, in principle, lead to the development of what can be called “disease digital twins” (Surra et al., 2025). Ecological modeling approaches have also been proposed and implemented to describe and study the complex dynamics of biological and biomedical systems (Aguadé-Gorgorió et al., 2024; Conte et al., 2023; Romano et al., 2021).

Within this broader landscape of systemic modeling approaches, the Energy Systems Language (ESL) provides a new quantitative framework that can represent the complex dynamics of biomedical systems. The proposed approach is already extensively used for studying dynamic patterns in ecological systems (Brown and Ulgiati, 2004; Conte et al., 2025; Hall and McWhirter, 2023; Maud and Cevolatti, 2004; Odum, 1971; 1995; 2002; Odum and Peterson, 1996; Odum et al., 2000), pointing out possible different scenarios and exploring their dependence on the values of variables and parameters. By encoding stocks, flows, and feedback loops within symbolic diagrams, ESL enables the construction of computational simulators that describe temporal evolution, regime shifts, and system-level responses to (controlled) perturbations (Odum and Odum, 2000).

In this work, the study of the Antigen-Specific Adaptive Immune Response (AISR) is presented as a typical example of complex, adaptive, systemic functioning at different time scales, suitable for demonstrating how ESL can quantitatively address and formalize complex biological dynamics. We developed an ESL model and a computational simulator that reproduces hallmark features of humoral immunity, including germinal-center dynamics, the establishment of long-lived plasma cells, sustained antibody secretion, and their long-term decline in the absence of antigen re-exposure. The model also addressed a critical problem in current modeling at the cell population level, namely, the formalization of spatial constraints in models of immune memory formation and plasma-cell-derived malignancies. This work demonstrates the potential of the Energy Systems Language to study these themes by integrating biological, biophysical and biomedical knowledge, hopefully boosting both interdisciplinary research and clinical practice.

## The energy systems language

### A symbolic quantitative language

Energy Systems Language (ESL) is a symbolic language originally derived from electronics and grounded in Systems Thinking theory. Initially developed for the analysis of ecological complexity, it traces its conceptual roots to the pioneering work by J. Forrester at MIT (Forrester, 1971). Significant improvements and formalization of ESL were introduced by systems ecologist Howard T. Odum, who developed a comprehensive symbolic and algebraic framework grounded in the Thermodynamics of open systems (Odum, 1994; Odum, 1996). ESL relies on the construction of systemic diagrams that describe the exchange, transformation, and management of resources within a system. These resources are made comparable through the use of a common energy-based unit, serving as a proxy for the various variables that define the system’s function. In the context of biological systems, an ATP-equivalent unit is commonly employed to standardize the accounting of all flows (Romano et al., 2021; Conte et al., 2023). Starting from these energetic diagrams, a system of coupled differential equations can be established, enabling the construction of quantitative computational simulators (Surra et al., 2025), capable of capturing complex behaviors of biomedical systems that emerge over time.

### Stock-flow diagrams

The fundamental elements of a systemic diagram are stocks, flows, and processes (interactions). The main symbols used (Odum and Odum, 2000) are shown in Figure 1.

**FIGURE 1:**
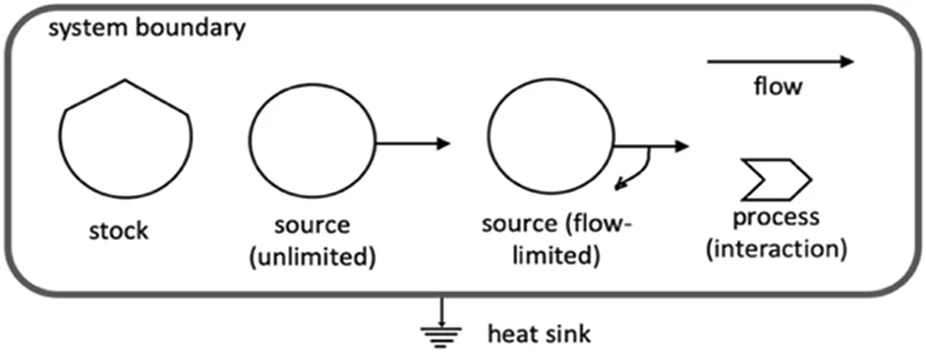
Main symbols used in stock-flow diagrams. In accordance with the second principle of Thermodynamics, a fraction of the available free energy is degraded and dissipated as heat in any physically occurring process; this is represented by the flow going to a heat sink.

#### Stocks

Stocks represent countable, extensive state variables *Q*_*i*_, *i* = 1,2,…,*n*, each containing a measurable quantity, e.g., of material, energy, information, cells, or even more abstract quantities such as space. At any given time, the set of stock values defines *the state* of the system. The selection of the stocks used in a systemic diagram depends on the hierarchical level of the intended analysis and the specific goals of the study. A stock may represent a physically localized component of the system as well as a distributed one (e.g., a stock of antibodies, or nutrients). It may also be necessary to represent the same kind of entity with multiple stocks—for example, separating a population of cells by location or developmental stage—based on their different role in the system’s dynamics. Although measurable, stocks should be chosen as the minimal set required to describe the state of the system for the analysis at issue. Any change in the system must correspond to a change in this *n*-tuple of state variables. The system’s state is therefore represented numerically within its phase space. Importantly, some stocks may appear clinically irrelevant in isolation but could play a critical role in the emergence of pathological systemic configurations.

#### Flows

A stock changes over time exclusively through its inflows and/or outflows. As such, a stock may function as a delay element, buffer, or shock absorber within the system. Flows represent the physical transfer of matter, energy, or information between stocks. In ESL, the system boundary in a diagram does not necessarily correspond to a physical boundary. Rather, it is chosen to clarify which inflows drive the systemic dynamics and which outflows represent the system’s outputs. For quantitative analysis, flows must be rendered analytically comparable. This is typically achieved by expressing flows in a common unit of energy. In biological systems, the ATP-equivalent may be used to standardize energy accounting, as it quantifies the energy required to support the generation or maintenance of a given flow.

#### Processes (interactions)

Processes encompass any mechanism that can alter the magnitude or the nature of a flow. During self-organization of a complex system, processes either work to maintain the system’s current state or enable its evolution toward new configurations. They mediate the exchange of resources between stocks and thus govern the system’s functionality. To be activated, a process requires input from other stocks or external drivers acting upon a flow. For this reason, processes are also referred to as interactions. It is important to note that the processes depicted in ESL models do not need to be an exhaustive catalog of known biochemical occurrences. Instead, they represent the network of systemic interactions that determine both the system dynamics and the actual accessible configurations. Consequently, the processes shown in stock-flow diagrams are not biological processes *per se*, but rather systemic symbols of effective interactions between the stocks. Each process effectively integrates the various biological, chemical, or molecular events influencing that interaction. While the specific molecular details may not be fully specified, the systemic representation fully captures their dynamic role within the model.

### System dynamics in the state space

The analytical model of a system is derived directly from the mathematical formulation of the flows represented in the diagram. For each of the stocks that define the system state, a corresponding balance equation is formulated in the general form:

$$\frac{dQ_{i}}{dt}=\sum_{\left\{in\right\}}J_{in,i}-\sum_{\left\{out\right\}}J_{out,i}=f_{i}\left(Q_{1},Q_{2},\ldots ,Q_{n}\right);i=1,\ldots n,$$ (1)

where the change in time of the *i*-th stock is given by the difference between the sum of all its inflows *J*_*in,i*_ and outflows *J*_*out,i*_, which is generally defined as a non-linear function of (in principle) all the stocks, *f*_*i*_. The diagram represents an analog circuit model of the (non-linear) relationships between stocks {*Q*_*i*_(*t*)}*, i = 1,…n* in the state space of the system. The coupled differential equations derived from it reflect the system’s actual dynamics. In this framework, a systemic model does not correspond to a single network, but rather to a class of structurally equivalent networks that share dynamic features. Thermodynamics provides the conceptual basis not only for the biomedical application of ESL diagrams, but also for its general application to complex systems, an aspect extensively treated in the foundational literature of the language (Odum, 1971; 1994; 1996; Brown and Ulgiati, 2004; Hall and McWhirter, 2023). Thermodynamics enters the ESL analytical description first of all in terms of energy conservation (first Principle), which motivates the use of energy as a common unit, and through the presence of the heat sinks in the diagrams, which allow the energy balance to be closed. The heat sink module represents (second Principle) the dispersal of available energy accompanied by an increase in entropy *S*. If *T* is the sink temperature, *dQ*/*dt* = *TdS*/*dt*. ESL algebra, described in the next paragraph, stems from the general linear form of the output of a single stock, as addressed by von Bertalanffy’s general systems theory (von Bertalanffy, 1968), where the outflows include both dispersal due to the second principle of Thermodynamics and the outflow of usable energy. Furthermore, the equations representing the system dynamics are linked to the thermodynamics of open systems, expressed for example in the Onsager relations (Onsager, 1931a; b; Hooyman and De Groot, 1955), which connect the evolution of open systems to the production of entropy. Onsager reciprocal relations in coupled multi-flow systems define the matrix of coefficients by linearly relating generalized flows and forces, describing how the effect of one force on a second flow equals the effect of the second force on the flow of the first, as extensively described, for example, by Odum (2007) and Brown (2025). Within this framework, in ESL the value of a stock acts as a generalized force on its outflow and flows are linearly related to driving gradients (Prigogine, 1955; Kondepudi and Prigogine, 2014; Katchalsky and Kedem, 1962). The linearity of the relations in coupled flow systems as described by Onsager is therefore related to the mutual interactions occurring at the level of ESL processes, as for example in predator-prey interactions, where the linearity of population growth rates leads directly to the multiplicative algebra described in the next paragraph.

Thermodynamic principles constrain the energetic representation and provide the physical foundation of ESL, while the multiplicative algebra provides a phenomenological systemic representation of interactions between stocks. However, the precise multiplicative form of all ESL interaction equations is not uniquely derived from first principles. To clarify this point, it is worth addressing the epistemological framework concerning the adjective “phenomenological” throughout the text. Phenomenological parameters are adjustable or measurable values in a scientific model that describes the observable behavior of a system. Values of the parameters are usually derived from the experimental observation of the predictions of the model at issue, like for example in the case of a best-fit procedure. In an ESL model, particularly for biomedical systems, the case is different, since the parameter values are directly measured or estimated in advance, based on data and behaviors that may have nothing to do with the system at issue. As for “phenomenological equations”, current epistemology addresses them as given and claimed to be true with only some explanations and justification, without derivation from first principles. Nevertheless, in the Physical Chemistry community, phenomenological equations are more specifically intended as linear mathematical relations used in non-equilibrium thermodynamics to connect thermodynamic driving forces (like temperature or concentration gradients) to their resulting transport responses, or flows (like heat or mass flow). In this sense, it is correct to use the expression “phenomenological equations” even in the case at issue for this work. However, as discussed above, the laws of Thermodynamics impose their constraints through both the first (energy conservation) and the second (entropy production) Principle. Moreover, ESL addresses the processes as the elements where mutual interaction of stocks takes place, as expressed by a multiplicative algebra.

### ESL algebra

In the presence of a process, the convergence of flows generates a flow as graphically represented in Figure 2a. Here, two flows *J*_*1*_ and *J*_*2*_ interact to produce a third flow *J*_*3*_. In this configuration, *Q* may represent, for example, a stock of organic molecules that, when processed owing to the proteins from stock *F*, are assembled into new species of organic molecules. More common representations of processes usually emphasize conserved quantities, ensuring that the total inflow (*J*_*1*_+*J*_*2*_) equals the total outflow (*J*_*3*_+*J*_*4*_). While this remains valid in ESL, the primary focus is on the relative actions of stocks (the system variables) participating in the process and captured through thermodynamically based generalized forces. Each flow in the diagram is therefore expressed as a product of stocks (*Q*⋅*F*), modulated by a coefficient *k* that reflects the overall effectiveness of the interaction. This representation captures the bidirectional dependence between interacting stocks. If the synthesis of a certain quantity flowing in *J*_*3*_ requires defined amounts of *Q* and *F*, a change in each stock value will proportionally affect all associated flows. This multiplicative formalism represents mutual causality and the complex feedback-driven dynamics of the system, beyond what additive energy balance equations alone can capture.

**FIGURE 2:**
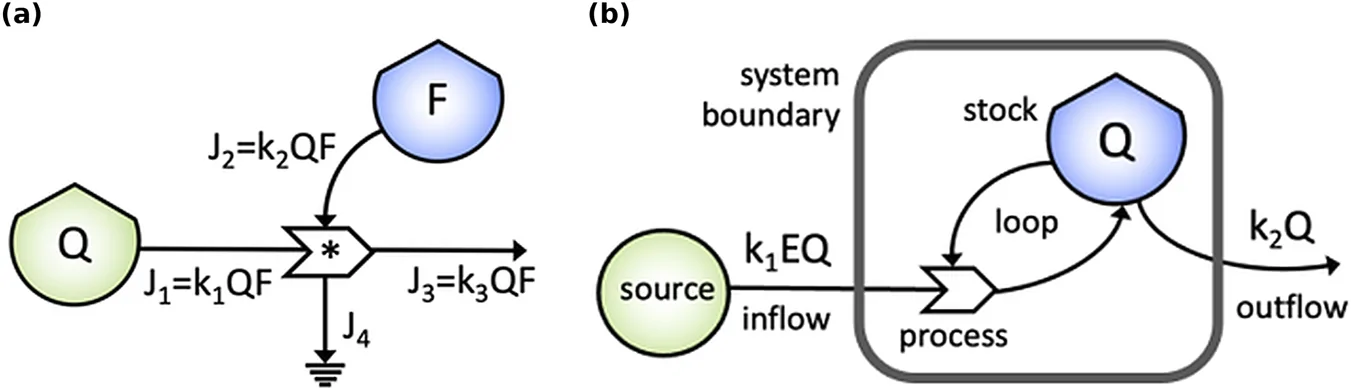
Symbolic representation of interaction. **(a)** Interaction of two flows from the stocks *Q* and *F*. **(b)** Stock-flow representation of a reinforcing feedback loop.

The primary driver of system dynamics in this framework is the mutual causality introduced by feedback loops. A feedback loop arises when a change in a stock affects flow(s) that, in turn, influence that same stock. A feedback loop is defined as a closed causal path involving at least one stock and one flow. When a change in a stock alters a flow that subsequently modifies the original stock, a feedback loop is present. These feedback interactions are central to the plasticity and adaptability observed in biological systems. Their non-linear and recursive nature allows complex temporal behaviors to emerge, which cannot be understood through linear, unidirectional cause-and-effect models. Such loops can involve multiple stocks and operate across different timescales, generating temporal hierarchies that introduce delays and additional complexity to the system’s function. Figure 2b illustrates a simple feedback system involving a single stock *Q*, subject to a reinforcing feedback, in which *Q* enhances its own inflow. The resulting dynamics depends on the balance between the feedback strength, expressed by *k*_*1*_, and the outflow rate, depending on *k*_*2*_.

The ESL multiplicative algebra expressing interactions has precedents outside ESL, notably in the seminal works of Lotka (Lotka, 1920; 1925; Volterra, 1926), who heuristically modeled predator-prey dynamics through coupled differential equations, with interaction terms formed by the product of population values. Such structural models do not emerge from explicit biophysical laws but from the systemic roles that stocks play in activating and regulating processes. Multiplicative algebras are also used to describe system dynamics in other scientific contexts. In Chemistry, the law of mass action follows a similar multiplicative rule, where the rate of a chemical reaction is proportional to the product of the activities (or concentrations) of the reactants. Even in this case, the validity of the law is rooted in Thermodynamics, since the fact that a reaction rate is proportional to the product of concentrations can be derived from the study of the frequencies of independent collisions in the description of gas kinetics, as developed first by Ludwig Boltzmann. As a matter of fact, Thermodynamics, along with statistical mechanics, is the framework capable of describing general dynamic behaviors and translating them into analytical equations, allowing one to explore the time evolution and the stability conditions of complexity.

When a system includes multiple processes and interacting stocks, the ESL modeling approach becomes increasingly valuable in accurately capturing its dynamics. The equation for a flow in this context does not describe the time derivative of a flowing quantity *per se*, but rather its dependence on the current system states defined by the values of the stocks. The functional dependence is not based on assumed physical laws or phenomenological coefficients (like, for example, in the case of diffusion phenomena) but is derived from the very systemic structure and from the known interactions. For example, in the case presented above, the flow resulting from the product of two stock values reflects the mutual influence that those stocks exert on one another’s evolution. At a higher level, this constitutes the feedback network that governs overall system dynamics. This conceptual framework in some sense parallels that of chaotic systems, where behavior cannot be traced back to local cause-and-effect chains, being instead characterized by trajectories and attractors in the state space (Strogatz, 2024). In our case, feedback loops play a role analogous to recursive equations in chaos theory, determining the system’s long-term behavior.

### Structural parameters

The selection of appropriate stocks and processes is fundamental to the system’s structure representation. The coefficients *k* represent structural parameters quantifying how the contributions of one or more stocks affect a process. They determine the intensity and responsiveness of flows, and by extension, the dynamics of the stocks themselves. For energy or matter flows, each coefficient *k* reflects the cumulative influence of all stocks contributing to a given inflow or outflow. These parameters are not associated with specific biophysical mechanisms but describe how a system component responds to changes in another. In cases where feedback is direct—such as in the proliferation of a population—coefficients can be directly estimated from known empirical rates, like fertility or mortality rates of biological populations. When feedback loops are more complex and involve multiple interacting stocks, the structural parameters are estimated from the outflow regimes of the stocks participating in the process at issue.

The ESL framework can, in principle, accommodate different types of research questions depending on the objectives of the analyses and data availability. This will be reflected case by case in the choice of model parameter setting and estimation strategies. In general, the values of the parameters depend on the biological and physiological features of the involved processes. In the case of biomedical systems, the structural parameters are (in principle) patient-dependent. The parameters that are not derivable from literature or direct measurements can either be constrained within biophysically plausible values (Romano et al., 2021; Conte et al., 2023) or optimized through data coming from specific case studies (Conte et al., 2025). In the latter case, the model becomes patient-specific, hence standard model validation procedures apply and the model might also show predictive features. For a general system model, as the one presented in this work, the degree of model credibility results from the simulator’s ability to qualitatively reproduce known system-level behaviors and response to perturbations of endogenous and exogenous components.

## Modeling biological systems with ESL

Applying ESL to biological systems involves the following structured workflow:

1. Definition of the system

The system definition must align with the intended goal of the dynamic study. ESL is not designed to offer a static depiction of an existing system but rather to provide a symbolic—and to some extent, abstract—representation of its behavior. It focuses on capturing the network of interconnections among those components that are actually relevant to the system’s dynamics.

2. Identification of main inputs, outputs, and system boundaries

The definition of inputs and outputs is informed by both the characteristics of the system and the objectives of the study. Notably, the boundary of an ESL-modeled biomedical system is not a fixed property of the system itself, but an epistemic construct tied to the modeling perspective. This boundary may vary depending on the hierarchical level of description, whether at the molecular, cellular, or population scale, or in the context of a specific disease scenario.

3. Selection of state variables (stocks)

The identification of system state variables is an operational decision, influenced by both biological relevance and modeling objectives. These are extensive variables (stocks) and define the phase space in which the system evolves.

4. Identification of flows between stocks and the environment

Flows represent the exchange of energy, matter, or information between stocks or across the system boundary. Their identification is essential for capturing the directionality and dynamics of system interactions.

5. Diagram construction

Diagrams are the central expressive tool of ESL. They offer a modular and adaptable representation of the system, amenable to implementation and modification in response to specific “what if” scenarios or biological conditions. Usually, this phase requires an intense collaboration between scientists coming from different disciplines (e.g., physicians, biologists, physicists and systems scientists). The amount of knowledge gathered has to be translated, following ESL rules, into a systemic diagram able to capture the complexity of the system at issue.

6. Formulation of coupled differential equations

Based on the diagrams, a system of coupled differential equations is directly defined using the multiplicative algebra of ESL. This requires initial values for stocks and, crucially, a set of parameters *k*. As discussed, these coefficients encapsulate the system’s dynamic properties, including the charge/discharge behavior of stocks and the efficacy of internal processes in altering flow patterns. Coefficients may be derived from empirical data, experimental measurements, or biologically informed estimations (see the Supplementary Material and the Structural parameters section).

7. Construction of a computational simulator

A computational simulator is built from the system of differential equations, using the defined initial conditions and structural parameters *k*. This simulator serves as a dynamic model of the system, capable of reproducing complex temporal behaviors of stocks at any relevant timescale. A local, one-parameter-at-a-time sensitivity analysis can be performed to evaluate model robustness; its scope and limitations are presented in the Discussion section. Additional technical details and case examples are provided in the Supplementary Material.

The construction of a simulator therefore relies on three distinct levels of knowledge:

- The architecture of the diagram defines the class of accessible behaviors. Rooted in the knowledge of the local details of the system functioning, it determines the dynamic features that must correspond to the observed reality. These depend on the systemic structure, and in particular on the network of feedbacks that ultimately governs the dynamics of the entire system. It also dictates which stocks participate and which processes rule the dynamics of the flows.
- The structural parameters define a specific trajectory within that class. The *k* coefficients determine the relative speed of the evolution of the stocks, the possible existence of threshold behaviors, and even the presence of latency times in a regime change. In biomedical systems they are, in principle, patient-dependent (Romano et al., 2021; Conte et al., 2023). They are not introduced *ad hoc* into the equations, but represent the relative strength of the processes that we want to highlight and identify. They are often known in advance through in-depth literature analysis and are in principle measurable with wet lab experiments (Surra et al., 2025). When a value is uncertain, suitable biological proxies are identified in order to estimate it as described in the Structural Parameters Section.
- Model calibration. A general assessment of a disease simulator may at a first stage proceed without a comprehensive knowledge of the full set of parameters, since a general simulation of a complex disease already opens the way to a novel representation of the disease onset and of its dynamic features. Such knowledge becomes mandatory for subsequent uses in medical practice. It is interesting to remark that the same epistemic route has already been developed in systems ecology, where the general study of a class of ecosystems can be followed separately by the contextualized study of a specific one (Brown and Ulgiati, 2004). Several iterations of this process lead to the complete set of variables and parameters.

Figures 3a–c illustrates different functional units commonly operating within complex biomedical systems, modeled using the ESL approach (Odum and Odum, 2000). Figure 3 shows recurrent biological motifs of competition, cooperation, and autocatalysis. These scenarios involve two populations, *Q*_*1*_ and *Q*_*2*_, in competition (Figure 3a) or cooperation (Figure 3b) for the use of a flow-limited resource *S*. Notably, in the case of competition, the stationary state of the system depends on the relative strength of internal reinforcing (proliferation) feedback loops, with only one population typically dominating and surviving. In contrast, in the cooperative scenario, a portion of the assets of each stock is invested in supporting the growth of the other population. Figure 3c presents a basic autocatalytic growth unit, depicting a self-organizing structure that utilizes recycling and production to reach a steady-state equilibrium. These functional units form the analytical bridge between the language introduced in Section *The energy systems language* and its practical application.

**FIGURE 3:**
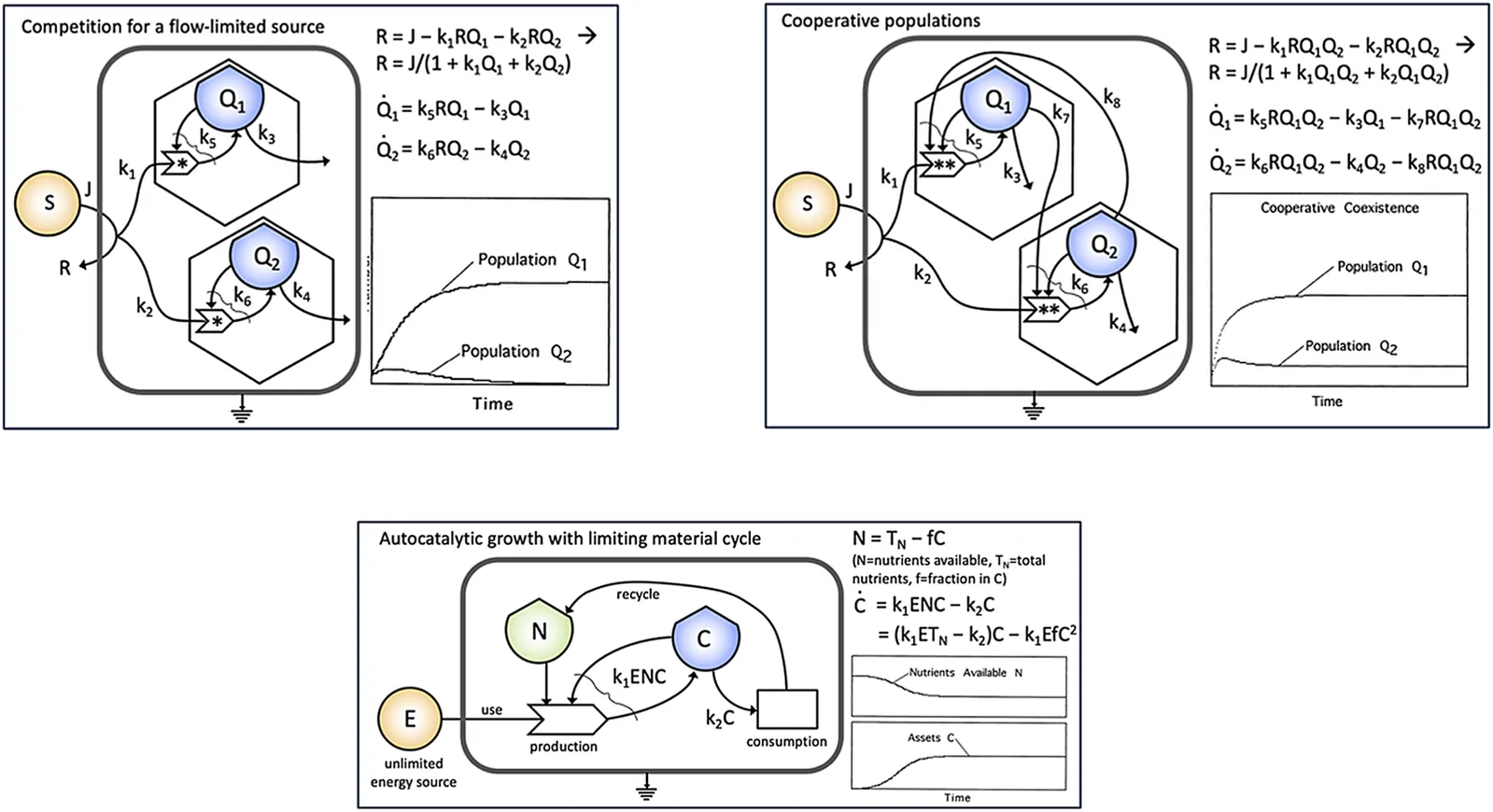
Functional units in ESL representations. The figures show minimal stock-flow models with corresponding differential equations and temporal evolution of the stocks: (Top left panel) two stocks competing for a flow-limited source; (Top right panel) two stocks cooperating for a flow-limited source; (Bottom panel) autocatalytic self-limiting unit.

## Application case study

### The adaptive response of the immune system using ESL

As a proof-of-concept, we built the ESL diagram of the antigen-specific adaptive immune response (AISR). This is a paradigmatic example of a well-known system, suitable for illustrating how ESL can quantitatively address and formalize the biological dynamics of complex biological systems at different time-scales. Starting from in-depth literature analysis, as described in the previous section, a stock-flow diagram describing the dynamics of the antigen-specific adaptive immune response is established (Figure 4). Each stock corresponds to a dynamically relevant immune cell population. Stocks are defined as follows: dendritic cells (*D*), naïve cells (*B*), antigens (*A*), antigen-specific *T*-cells (*T*), antigen presented by specific *T*-cells (*Q*_*1*_), antigen presented by specific dendritic cells (*Q*_*2*_), antigen-specific *B*-cells, including *B*-memory (*Q*_*3*_), plasmablasts and short-lived plasma cells (*Q*_*4*_), long-lived plasma cells (*Q*_*5*_), immunoglobulins able to recognize a specific antigen (*Q*_*6*_), available space in the bone niche (*Q*_*7*_), managed by stromal cells able to mediate plasma cell homing in the bone marrow. Stocks of resources (*E*) reflect metabolic support systems. Here, *Q*_*1*_ and *Q*_*2*_ do not represent distinct cell types *per se*, but rather effective systemic stocks quantifying antigen presentation mediated through *T*-cell cognate help and dendritic cell priming, respectively. In traditional immunology, *T*-cells are not classical professional antigen-presenting cells; however, in the current diagram, *Q*_*1*_ is an effective systemic stock representing the functional state of *T*-cell-mediated cognate help. Specifically, *Q*_*1*_ abstracts the multi-step pathway wherein *T*-cells are activated by antigen and subsequently display/transmit activation signals (e.g., CD40L and cytokine secretion) to *B*-cells. Likewise, *Q*_*2*_ captures the primary dendritic cell processing and presentation pathway. Representing these as distinct systemic flows J1’ and J2’ preserves the indispensable biological logic of adaptive humoral immunity: *B*-cell activation and clonal expansion (*Q*_*3*_) strictly require both antigen-priming inputs (*Q*_*2*_) and helper *T*-cell signals (*Q*_*1*_).

**FIGURE 4:**
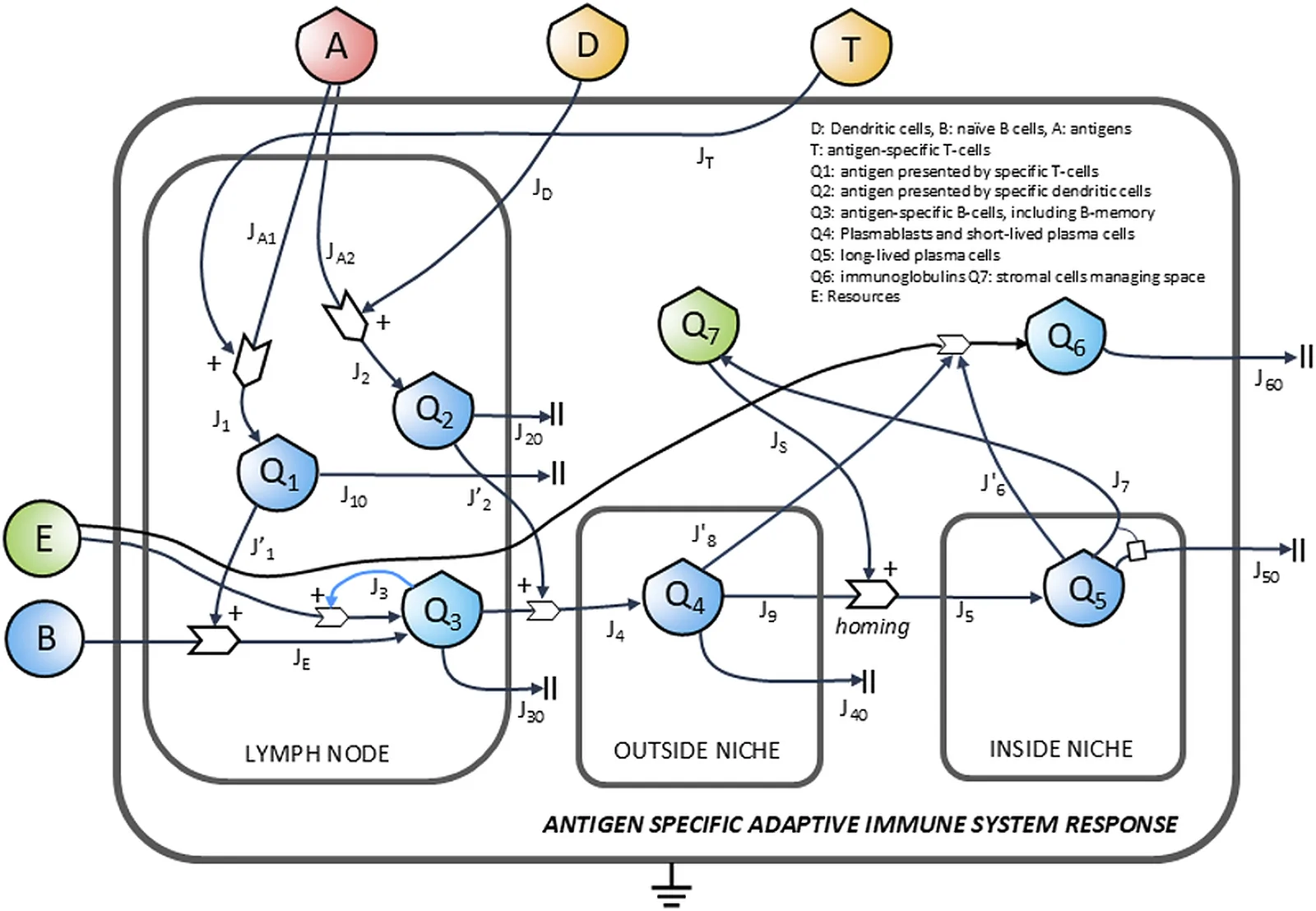
Stock-flow diagram of the antigen specific adaptive immune system response. Energy Systems Language representation of the dynamics of the Antigen-Specific adaptive Immune Response.

The choice of initial values at the time of simulation *t*_*0*_, based on experimental observations in humans and mice, is reported in Supplementary Table S1 in the Supplementary Material.

In the model, the *J*_*i*_ terms represent flows of matter, except for *J*_*S*_ and *J*_*7*_, which are defined as flows of units of available space. The *J*_*i*_ *’* terms correspond to *control flows* and rule-specific processes. Accordingly, they are not treated as stock outputs in the equations, as their energetic contribution is negligible relative to the primary flows (see also Supplementary Material). Symbols with two lines at the end of a flow indicate the process of apoptosis or natural degradation for the respective stock. The small square present on the flow *J*_*5A*_ represents a probe for the flux *J*_*7*_: when the stock *Q*_*5*_ decreases its value by one unit, the probe signals the flow *J*_*7*_ to replenish the available stock *Q*_*7*_ by one unit. The diagram depicts the dynamics of antigen-specific adaptive immune response (AISR) as a stock-flow system structured across three sub-systems, biologically equivalent to the lymph node and two niches: the outside niche—peripheral blood and the inside niche—bone marrow. Within the lymph node sub-system, antigen (*A*) interacts with stocks *Q*_*1*_ and *Q*_*2*_, driving the generation of *Q*_*3*_ (biologically equivalent to *B*-memory cells), which undergoes selection and differentiation into *Q*_*4*_ (biologically equivalent to short-lived plasma cells). The latter produces the output stock *Q*_*6*_, while part of its flow is directed toward the inside niche sub-system. In this second niche, entry and persistence of *Q*_*5*_ (biologically equivalent to long-lived plasma cells) are regulated by spatial constraints represented by *Q*_*7*_ (space). Once established, *Q*_*5*_ sustains the long-term production of *Q*_*6*_ (antibodies) through the secretion of antigen-specific antibodies, without further need of antigen stimulation. The metabolic support stock (*E*) contributes to several processes across the system. This dual-niche organization highlights the transient dynamics of *Q*_*5*_ in the lymph node versus the long-term maintenance of *Q*_*5*_ and *Q*_*6*_ in the bone marrow, capturing both short- and long-term phases of the adaptive response. Under healthy conditions, the bone marrow inside the niche provides limited space and trophic support, ensuring a balanced turnover of plasma cells and a stable output of antigen-specific antibodies.

Each stock and process in Figure 4 represents an effective systemic abstraction that aggregates multiple underlying cellular and molecular steps. For instance, *Q*_*1*_ and *Q*_*2*_ aggregate complex antigen processing, MHC presentation, TCR/costimulatory binding, and localized cytokine secretion into unified systemic presentation flows (*J*_*1*_ *, J*_*2*_). To maintain a minimal state-space required for long-term multi-timescale simulation, certain micro-scale processes—such as explicit somatic hypermutation pathways, affinity maturation sub-lineages, Treg suppression networks, and micro-anatomical cell migration dynamics—were deliberately excluded. These fine-grained mechanisms are implicitly embedded in the overall process efficiency coefficients *k*_*i*_. A complete biological mapping, including the entities represented, aggregated processes, supporting literature evidence, and explicit exclusions for every stock and flow in Figure 4, is provided in Supplementary Table S3.

### Energetic conversions of stocks and flows

All stocks, flows, and processes were expressed using a common proxy unit, representing the energy embedded, transmitted, and used, respectively, during the system operation. The proxy unit was expressed as ATP-equivalent units, as previously described (Romano et al., 2021; Conte et al., 2023); *ATPeq* serves as a measure of the thermodynamic free energy stored in cell stocks. This choice allowed us to calculate each parameter of the system based on stocks and characteristic times of the flows derived from the literature, without further need for experimental data.

One ATPeq corresponds to the free energy made available by the hydrolysis of one ATP molecule, 1 *ATPeq*≡10^–19^ *J*. Cell stocks (*Q*_*1*_-*Q*_*5*_, and the source stocks *B*, *D* and *T*) are converted through the ATP investment required to build a single cell, 1 cell≡5·10^10^ *ATPeq*≡5·10^−9^ *J* (Conte et al., 2023). The stock of niche space *Q*_*7*_ is converted with the same factor, because one unit of space is defined as the volume released by one long-lived plasma cell, as encoded by the probe on flow *J*_*5A*_; this conversion serves as an accounting modelling tool that renders the niche constraint commensurable with the selected cell stocks. The immunoglobulin stock *Q*_*6*_ is converted through the biosynthetic cost of peptide-bond formation, taken as 4 ATP per residue with a mean residue mass of 110 *g/mol*, which yields 2.19·10^22^ *ATPeq* per Gram of protein. Antigen stocks are converted through the same protein-cost route, which for a protein antigen of 50 *kDa* gives 1.8·10^3^ *ATPeq* per molecule. See the Supplementary Material section for mathematical details of the calculations.

### Dynamics of the antigen-specific adaptive immune response

The systemic diagram in Figure 4 is translated into a set of coupled ordinary differential equations (ODEs) that mathematically describe the dynamic behavior of each immune component over time, as in Equation 1. These equations are integrated over time to simulate the progression of the Adaptive Immune Response under varying conditions. Initial values for the variables of the model are presented in Supplementary Table S1 (see Supplementary Material); below, the full set of differential equations for the model is shown:

$$\frac{dQ_{1}}{dt}=k_{1}AT-k_{1A}Q_{1}$$

$$\frac{dQ_{2}}{dt}=k_{2}AD-k_{2A}Q_{2}$$

$$\frac{{dQ}_{3}}{dt}=k_{3}EQ_{3}+k_{B}BQ_{1}-k_{4}Q_{3}Q_{2}-k_{3A}Q_{3}$$

$$\frac{{dQ}_{4}}{dt}=k_{4}Q_{2}Q_{3}-k_{9}Q_{4}Q_{7}-k_{4A}Q_{4}$$ (2)

$$\frac{{dQ}_{5}}{dt}=k_{5}Q_{4}Q_{7}-k_{5A}Q_{5}$$

$$\frac{dQ_{6}}{dt}=k_{8}EQ_{4}+k_{6}EQ_{5}-k_{6D}Q_{6}$$

$$\frac{{dQ}_{7}}{dt}=k_{5A}Q_{5}-k_{5}Q_{7}Q_{4}$$

$$\frac{dE}{dt}=0$$

### Simulation setup, model constraints and numerical implementation

The system of Equation 2 is numerically integrated through simulation from a series of initial values calculated using ESL rules (see Supplementary Material) on data from the literature, for each of the stocks of cell populations and external system resources. Equations were integrated with the implicit Runge-Kutta method, as implemented in the *solve_ivp* routine of the *SciPy* library in Python, with adaptive step size and default tolerances (relative 10^–3^, absolute 10^–6^). All simulation codes used in this study were developed in Python and are openly available on GitHub at: https://github.com/federicosurra-venice/AISR_SMAC.git. The repository includes the scripts required to reproduce the results presented in this work, together with a sensitivity analysis of the model. Trajectories were sampled on uniform grids of 100, 1000 and 10^4^ points over the three horizons of 10 days, 1 year and 80 years, respectively. The naïve *B*-cell pool (*B*), the dendritic cell pool (*D*) and the antigen-specific *T*-cell pool (*T*) are held constant throughout each simulation at the values reported in Supplementary Table S1 (see Supplementary Material), on the assumption that the antigen-specific response consumes a negligible fraction of these pools over the timescales considered. The metabolic resource stock *E* is a normalized forcing function; its role is to make explicit which processes draw on metabolic resources, while its absolute magnitude is absorbed into the corresponding coefficients.

The antigen stock is held at *A* = 2·10^6^ antigen units for a priming interval of 1 day, and is set to *A* = 0 thereafter; antigen clearance is therefore modelled as instantaneous at the end of the priming interval, and no antigen re-exposure occurs. Because the priming interval is short compared with every other timescale of the model, what the simulations display is the free evolution of the system once the stimulus has been removed. The analogy with the impulse response of an electronic circuit is intrinsic to the very nature of ESL, which derives its symbolic and algebraic apparatus from circuit analysis (Odum, 1971).

The related simulator can be operated across different time scales, ranging from days, months, to years, as shown in Figure 5. Simulations are performed with parameter settings as in Table 1, including 5% uncertainty bands on all parameters. The bands represent an exploratory parameter-perturbation envelope rather than a statistical confidence or credible interval and are varied independently and deterministically one at a time. On the time scale of days (Figure 5A), antigen presentation occurs via interaction between stocks A , D , and T , feeding the presentation stocks *Q*_*1*_ and *Q*_*2*_. This dual signal drives the activation and expansion of *Q*_*3*_ in the germinal center reaction (range 0–5 days, antigen-specific *B*-cells, including activated germinal center *B*-cells and memory *B*-cells). *Q*_*3*_ then undergoes selection and differentiation to form *Q*_*4*_ (short-lived plasma cells), which initiates early antibody secretion (Q6). Afterwards, *Q*_*4*_ rapidly declines and the synthesis and secretion of *Q*_*6*_ (antigen-specific antibodies) is performed by the rising stock *Q*_*5*_ (long-lived plasma cells). On the time scale of 1 year (Figures 5B,E), when the antigen is cleared, *Q5 grows to stationary states associated* with stable states of *Q*_*6,*_ explaining the persistence of antibodies for years.

**FIGURE 5:**
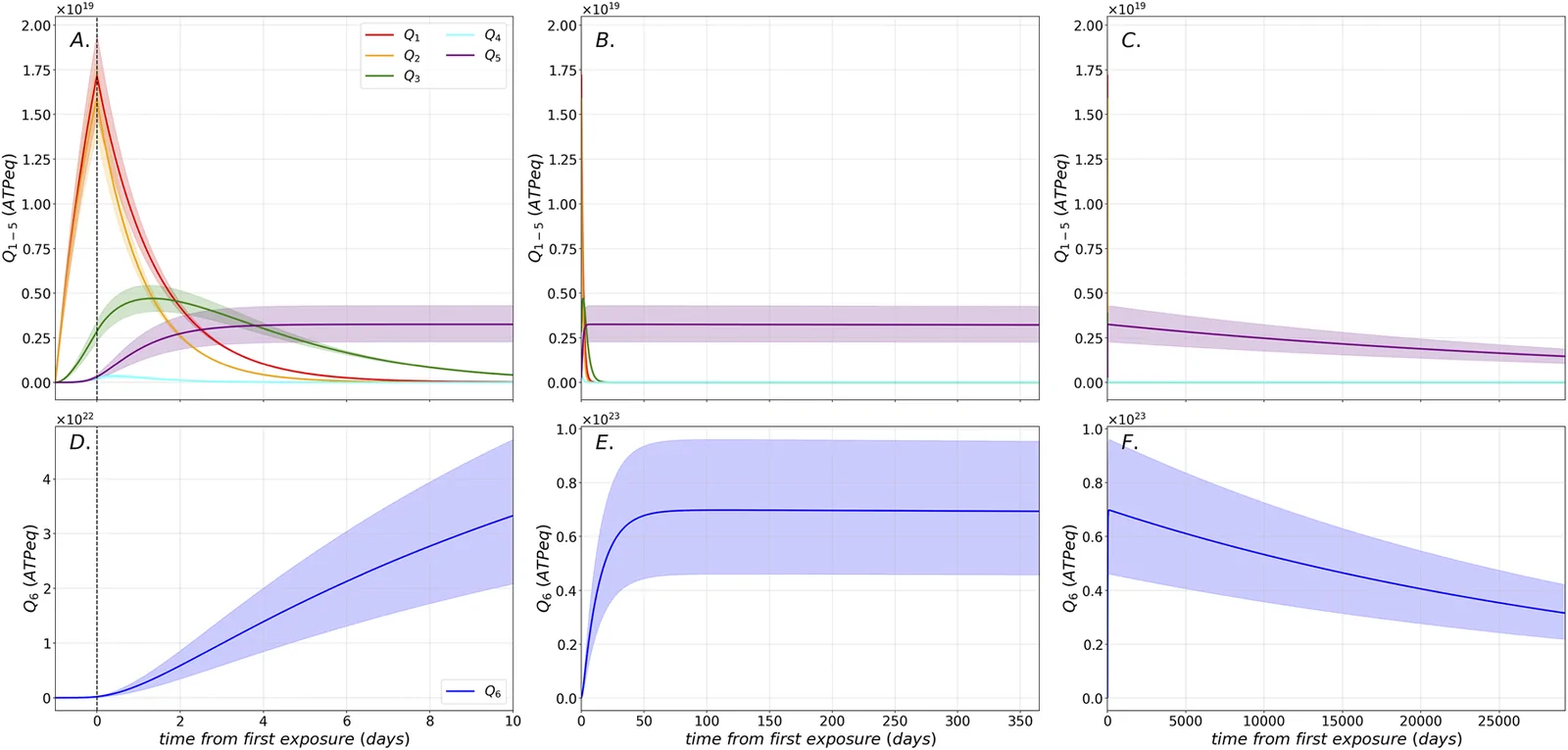
Dynamics of the specific adaptive immune response at daily, monthly and decadal timescales. Simulations are performed with parameter settings as in Table 1 for **(A,D)** 10 days, **(B,E)** 1 year, **(C,F)** 80 years, including 5% uncertainty on all parameters (see text). Figures show the cell stocks *Q*_*1*_ (red), *Q*_*2*_ (yellow), *Q*_*3*_ (green), *Q*_*4*_ (cyan), and *Q*_*5*_ (purple), and the antibody stock *Q*_*6*_ (blue, bottom) in *ATPeq* units with the associated uncertainty in the trajectories. **(A,D)** Show the fast transient response of the immune system to first antigen exposure. **(B,E)** show its stationary behavior and the persistence of long-lived plasma cells and *A*-specific antibodies, while **(C,F)** show the decay of the long-lived plasma cells and *A*-specific antibodies over long time scales.

**TABLE 1:** Values of flows and *k* parameters in the ODEs system.

Flow	Description	Flow value	k (Δt = 1 day), native units	Conversion factor applied	k (Δt = 1 day), ATPeq units
J_A1_	Flow of Ag that crowds Q_1_ (Abbas et al., 2015)	(10^6^–10^8^) [Ag/day]	—	× c_Ag_	(1.8·10^9^–1.8·10^11^) [ATPeq day^–1^]
J_A2_	Flow of Ag that crowds Q_2_ (Abbas et al., 2015)	(10^6^–10^8^) [Ag/day]	—	× c_Ag_	(1.8·10^9^–1.8·10^11^) [ATPeq day^–1^]
J_E_	Affinity selection: T-Ag cells entering B-Spec formation (Jones et al., 2015)	0.85 B [day^–1^]	k_B_ = 10^–9^ [day^–1^ cell^–1^]	1/c_cell_	k_B_ = 2.0·10^−20^ [day^–1^ ATPeq^−1^]
J_1_	Flow of T-Ag cells that crowds Q_1_ (Lanzavecchia et al., 1988)	240 [cells/day] = 1.2·10^13^ [ATPeq/day]	k_1_ = 28·10^−9^ [day^–1^ Ag^−1^]	1/c_Ag_	k_1_ = 1.5·10^−11^ [day^–1^ ATPeq^−1^]
J_2_	Flow of D-Ag cells that crowds Q_2_ (Conte et al., 2023)	240 [cells/day] = 1.2·10^13^ [ATPeq/day]	k_2_ = 6·10^−6^ [day^–1^ Ag^−1^]	1/c_Ag_	k_2_ = 3.3 · 10^−9^ [day^–1^ ATPeq^−1^]
J_3_	Proliferation rate of B-Spec cells (Macallan et al., 2005)	—	k_3_ = 0.019 [day^–1^ E^–1^]	invariant	k_3_ = 0.019 [day^-1^ E^-1^]
J_4_	Plasma cells flow in the outside niche (Brynjolfsson et al., 2018)	0.50·Q_3_/5.5 weeks	k_4_ = 2·10^−9^ [day^–1^ cell^–1^]	1/c_cell_	k_4_ = 4.0·10^−20^ [day^–1^ ATPeq^−1^]
J_5_	Plasmablasts becoming plasma cells of the inside niche (Aaron and Fooksman, 2022)	0.80·Q_4_/4 h	k_5_ = 4.8 · 10^−9^ [day^–1^ S^−1^]	1/c_S_	k_5_ = 9.6·10^−20^ [day^–1^ ATPeq^−1^]
J'_6_	Rate of Q_5_ cells secreting Ig (Bromage et al., 2009)	control	k6 = 3.4 · 10^−9^ [g cell^–1^ day^–1^]	× c_Ig_/c_cell_	k_6_ = 1.5·10^3^ [day^–1^]
J_7_	Flow of space (bone marrow)	—	—	—	—
J'_8_	Flow of plasmablasts that crowd the stock of Ig (Bromage et al., 2009)	control	k_8_ = 3.4 · 10^−9^ [g cell^–1^ day^–1^]	× c_Ig_/c_cell_	k8 = 1.5·10^3^ [day^–1^]
J_9_	Plasmablasts participating in the homing process (Cassese et al., 2001)	0.03·Q_4_/3 days	k_9_ = k_5_	1/c_S_	k_9_ = k_5_
J_S_	Flow of space (homing)	—	—	—	—
J_1A_	Natural apoptosis rate for T-Ag cells (De Boer et al., 2003)	0.50·Q_1,ss_/1 day	k_1A_ = 0.693 [day^–1^]	invariant	k_1A_ = 0.693 [day^–1^]
J_2A_	Natural apoptosis rate for D-Ag cells (Matsue et al., 1999)	0.50·Q_2,ss_/20 h	k_2A_ = 0.85 [day^–1^]	invariant	k_2A_ = 0.85 [day^–1^]
J_3A_	Apoptosis rate of B-Spec cells (Brynjolfsson et al., 2018)	0.70·Q_3,ss_/3 days	k_3A_ = 0.4 [day^–1^]	invariant	k_3A_ = 0.4 [day^–1^]
J_4A_	Natural apoptosis rate of plasmablasts, outside niche (Cassese et al., 2003)	0.97·Q_4,ss_/7 days	k_4A_ = 0.33 [day^–1^]	invariant	k_4A_ = 0.33 [day^–1^]
J_5A_	Apoptosis rate of cells in the inside niche (Andraud et al., 2012)	0.10·Q_5,ss_/10 years	k_5A_ = 2.7 · 10^−5^ [day^–1^]	invariant	k_5A_ = 2.7 · 10^−5^ [day^–1^]
J_6D_	Natural degradation rate of Ig (Andraud et al., 2012)	0.50·Q_6,ss_/10 days	k_6D_ = 0.07 [day^–1^]	invariant	k_6D_ = 0.07 [day^–1^]
J'_1_	T-Ag cells controlling the affinity selection process	control	—	—	—
J'_2_	D-Ag cells controlling the affinity selection process	control	—	—	—

Conversion factors: c_cell_ = 5·10^10^ ATPeq, per cell (≡5·10^−9^ J; Conte et al., 2023); c_S_ = c_cell_, since one niche slot is defined as the volume released by one long-lived plasma cell; c_Ig_ = 2.19·10^22^ ATPeq, per Gram of immunoglobulin (4 ATP, per peptide bond, mean residue mass 110 g/mol), equivalently 5.3·10^3^ ATPeq, per IgG molecule; c_Ag_ = 1.8·10^3^ ATPeq, per antigen molecule (50 kDa); 1 ATPeq≡10^–19^ J. Simulations were run with the rounded values k1A = 0.7 and k2A = 0.9 days–1, in place of the 0.693 and 0.85 derived from the corresponding literature half-lives.

Simulations were performed across a wide range of timescales:

- from the first day of antigen exposure, describing transient states of response within 10 days;
- for 1 year since antigen exposure, showing persistence and stationarity of Long-lived plasma cells (LLPC) and antibodies;
- for 80 years, which shows the long-term decline of antibodies in the absence of antigen re-exposure.

The results are compatible with experimental observations of the extinction of short-lived plasma cells within 10 days of the first antigen exposure and persistence of long-lived plasma cells over decades (Schwarz et al., 2017). On the timescale of decades (Figure 5C), *Q*_*5*_ and *Q*_*6*_ exponentially decay over time according to their intrinsic baseline loss rate *k*_*5A*_, illustrating the natural intrinsic turnover of antigen-specific long-lived plasma cells and immunoglobulins in the absence of re-exposure. While this long-term simulation captures the baseline loss of humoral memory over decades, it represents a structural baseline decay driven by constant kinetic parameters rather than an explicit mechanistic model of active immunosenescence.

The simulations reproduce patterns that align with empirical knowledge related to the response to primary vaccinations or to certain infections (Bagheri-Jamebozorgi et al., 2014). A subset of plasma cells persists for decades, continuously secreting antibodies even in the absence of antigen re-exposure, explaining the maintenance of immunity (Amanna and Slifka, 2010). Expressing the stocks in a common energetic unit makes it possible to compare compartments of different biological nature on the same level. At steady state the antigen-specific immunoglobulin stock *Q*_*6*_ holds 6.69·10^22^ *ATPeq* (6.7 *kJ*), against 3.2·10^18^ *ATPeq* (0.32 *J*) held by the long-lived plasma-cell population *Q*_*5*_ that sustains it: in energetic terms, under the present ATP-equivalent accounting method, the inventory associated with the antibody population is about four orders of magnitude larger than that of the long-lived plasma cells that sustain it. A set of estimations for the flows and the structural parameters in Equation 2 is shown in Table 1. A local, one-parameter-at-a-time (OPT) sensitivity analysis of the structural parameters was performed to evaluate regime stability around the baseline system-level trajectory. Technical details and calculations are provided in the Supplementary Material.

The existence of an upper bound on the value of the long-lived plasma-cell population seen in Figure 5 is an explicit modelling assumption. In the model, the bone-marrow niche offers a finite number of survival slots: flow J_5A_ returns to *Q*_*7*_ exactly the space released by each cell lost from *Q*_*5*_, so that Equation 2 imply dQ7/dt *= –dQ*_*5*_ */dt* and the sum *Q*_*5*_+*Q*_*7*_ = *Q*_*7,MAX*_ is conserved. The saturation of *Q*_*5*_ at high antigen load is therefore a structural property of the representation of niche space.

The logistic levels of *Q*_*5*_ and *Q*_*6*_ and the rate of the long-term decline are set by individual parameter choices regarding the value of *Q*_*7,MAX*_, together with the apoptosis rates of the plasmablast and plasma-cell compartments: the decays of stocks *Q*_*5*_ and *Q*_*6*_ over the decadal horizon are in fact governed by their related degradation rates *k*_*5A*_ and *k*_*6D*_. The longer-timescale simulations (50–80 years) should accordingly be read as the long-term consequence of these specified constant loss parameters.

The processes connecting stocks of cell populations act as effective systemic interactions with respect to the underlying biochemical mechanisms: antigen presentation (*J*_*A1,2*_), the result of affinity selection (*J*_*E*_) and the homing processes (*J*_*8,9*_) are, in this regard, each represented by a single flow. The model is therefore informative about the systemic organization of the antigen-specific adaptive immune response by construction and does not focus explicitly on singular, specific molecular aspects.

### Threshold behavior of the antigen-specific adaptive immune system response

As previously stated, in the model the number of LLPCs is limited by the combination of natural apoptosis rates (*J*_*5A*_ *, J*_*4A*_) and niche constraints *Q*_*7*_. Thus, *Q*_*7*_ acts as the structural constraint that shapes immune memory formation. It should be underlined that the space stock does not represent a physical storage of free energy: its role is systemically essential since it acts as a control on the homing process, and this is substantiated by the balance between the stock of units of space and the stock of long-lived plasma cells. This is an example of the flexibility of ESL in representing the quantities that are relevant to the overall functioning of a system. The niche constraint generates a threshold-like behavior for the stationary values of LLPCs in response to increasing antigen load ranging from 10^5^ to 9·10^7^ antigen units. Figure 6A shows the relationship between the stationary values of *Q*_*5*_ (LLPCs), normalized to their respective maximum value *q*_*5*_ = *Q*_*5*_/*Q*_*5,MAX*_, as a function of the normalized antigen load a = A/AMAX, for simulations running over a time span of 30 days. Figure 6B shows the relationship between the normalized *Q_5_* stock as a function of time from first antigen exposure over the same simulation time span. Equation 3 represents a sigmoid law between normalized stationary values of LLPC in response to increasing antigen load:

$$q_{5,ss}\left(a\right)=\frac{\alpha }{1+e^{-b\left(a-c\right)}}+\delta$$ (3)

with *b*≃33. Simulations reproduce a minimum response (*q*_*5*_ *< 10%*) for normalized antigen load between 10^−3^ and 10^−2^. Increasing the antigen load up to a threshold *Q*_*A*_ *th* = 7.26·10^6^ antigen units = 1.32·10^10^ *ATPeq*, LLPCs sharply reach their stationary maximum value. This value sets the limit to immune memory formation for *a>*10^−1^.

**FIGURE 6:**
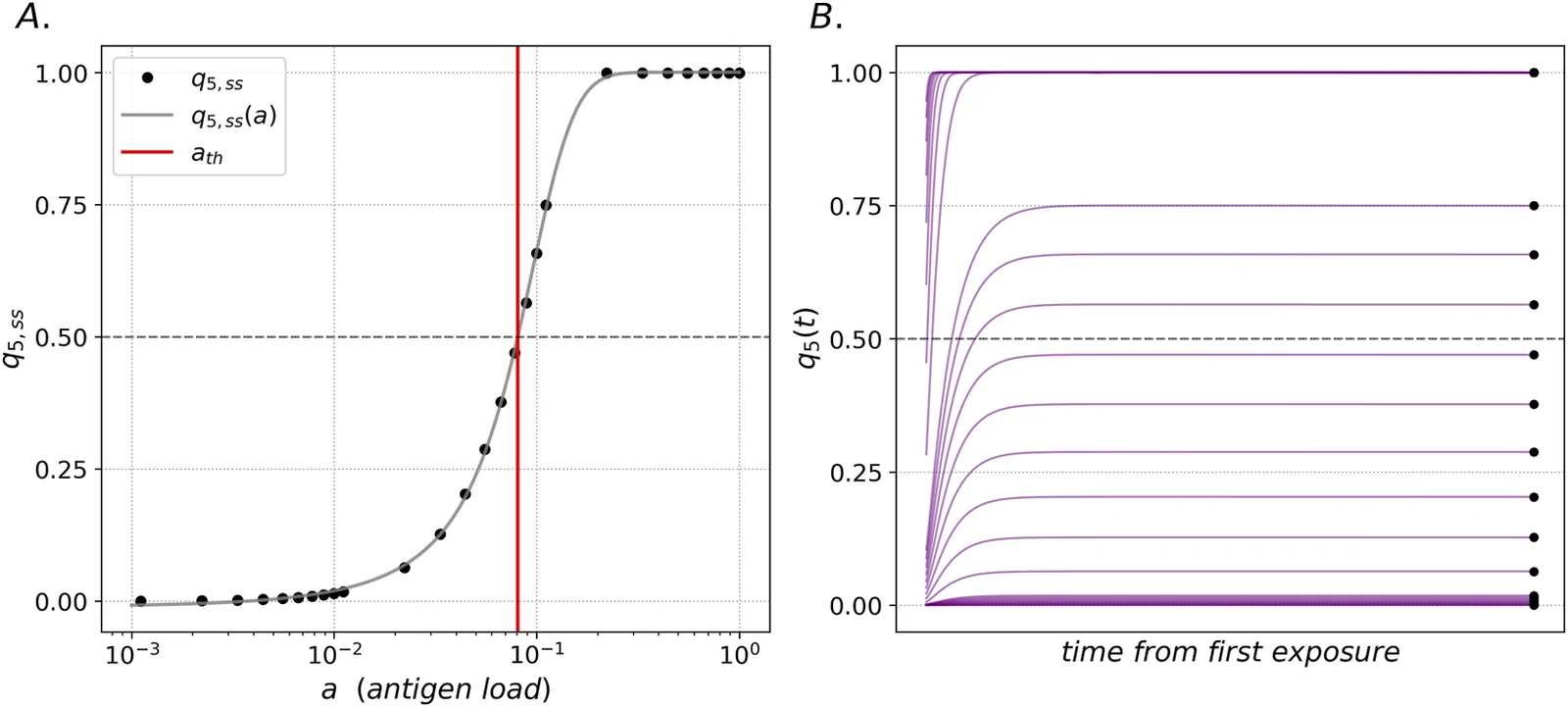
Threshold behavior of the immune response. **(A)** Stationary states of the immune system response showing the normalized *Q*_*5*_ stock (*q*_*5,ss*_) as a function of the normalized antigen load (a). **(B)** Dynamics of the immune system response showing the normalized *Q*_*5*_ stock (*q*_*5*_) as a function of time t, for a 30-day simulation with parameter settings from Table 1 and for increasing antigen load A from 10^5^ to 9·10^7^ units (purple curves), 1.8·10^8^–1.6·10^11^ *ATPeq*; black dots identify the stationary states. The grey curve is the sigmoid function describing the states $q_{5,ss}\left(a\right)=\frac{\alpha }{1+e^{-b\left(a-c\right)}}+\delta$ , with parameters *α* = 1.10, *b* = 32.6, *c* = 0.075, *δ* = −0.10 and root mean square error RMSE = 0.44%. The red vertical line indicates the estimated threshold of the antigen load required for the response at the maximum capacity.

A minimum antigen load is required to activate and sustain the proliferation of *Q*_*3,*_ driving the LLPC formation (*Q*_*5*_). The finite availability of space in the bone marrow niche (*Q*_*7*_) imposes an upper bound on the number of persistent LLPC. As *Q*_*7*_ saturates, additional LLPCs cannot be maintained, producing the observed behavior. The model reproduces a steep response once the antigen load threshold is crossed, with plasma cell population expansion saturating at the maximum carrying capacity of the niche, a typical hallmark of systemic complexity: even if antigen load is high, LLPCs cannot expand indefinitely because spatial resources in the bone marrow are finite. This ensures that immune memory stabilizes at a plateau. This pattern reproduces configurations of finite acquired immunity ensured by the long-term persistence of LLPCs, secreting antigen-specific antibodies.

## Discussion

Energy Systems Language is an approach that develops its analytical aspect within a more general framework, that of Systems Thinking, as set out for example by Donella Meadows (Meadows, 2008). Starting from the basic Systems Thinking concepts—in particular, mutual interconnectedness, feedback networks, leverage points—ESL develops quantitative analytical aspects that substantiate Meadows’ Systems Thinking, with significant potential in the application of systemic approaches. The ESL framework enables the description of complex biological functions and the identification of emergent features in biomedical systems. Grounded in established biological knowledge, the approach introduces explicit time dependence in the representation of system states. The use of ESL formalism represents an epistemological innovation in the study of biomedical complexity. The dynamic descriptions offered by stock-flow diagrams, subsequently translated into sets of differential equations, generate new insights by representing interactions effectively, integrating prior knowledge of biochemical details at the local level. In fact, the processes depicted in ESL are not intended to represent biological mechanisms *per se*. Instead, they are systemic processes designed to capture temporal dynamics—what happens, and what will happen, to the variables characterizing the system (Romano et al., 2021; Conte et al., 2023; Surra et al., 2025). Crucially, the form of the equations governing system dynamics is constrained by the thermodynamics of open systems: the multiplicative algebra is a phenomenological representation of systemic interactions in ESL, while the energetic accounting for the cell populations ensures that heterogeneous stocks are expressed in a common unit. Operationally, the multiplicative ESL algebra, which originated in systems ecology (Odum, 1994) and is also applied in cancer ecology (Aguadé-Gorgorió et al., 2024), is used to describe how flows arise from the network of interconnections among system elements. The form of these equations is thus rooted in the Thermodynamics of open systems and derived directly from the symbolic structure of systemic diagrams.

We presented the case of AISR, where dynamical simulation of cell population evolution and antibody secretion was determined at various time scales, describing acquired immunity. The results demonstrate how ESL can reproduce adaptive and complex behavior in biological systems, starting from a systemic structural analysis of stocks, flows, and feedback mechanisms. While our long-duration (80-year) simulations illustrate the slow, persistent clearance of *Q*_*5*_ and *Q*_*6*_, this dynamic is primarily governed by the intrinsic loss rate (*k*_*5A*_) under constant parameter conditions. To frame these long-term dynamics fully as a mechanistic model of immune ageing (immunosenescence), explicit age-dependent functions would need to be incorporated—for instance, modeling the progressive age-related degradation of the bone marrow microenvironment (e.g., dynamic decay of *Q*_*7*_ capacity) or time-variant apoptosis rates *k*_*5A*_(*t*). In its current formulation, the model serves as a healthy baseline framework upon which such age-dependent or pathological perturbations can be explicitly introduced.

A local, one-parameter-at-a-time (OPT) sensitivity analysis of the structural parameters was performed. While the approach evaluated parameter robustness and regime stability around baseline trajectories, it might not fully capture non-linear parameter interdependencies and global parameter space interactions in multiplicative flux terms (of the form *J*_*ij*_ *= k*_*ij*_ *Q*_*i*_ *Q*_*j*_). To strengthen the applications of this framework in patient-specific problems, future studies might incorporate stability analysis (e.g., Conte et al., 2023) and variance-based Global Sensitivity Analysis (GSA) (e.g., Rampoldi et al., 2026) to systematically map high-dimensional parameter interactions across disparate dynamic regimes.

The ESL model qualitatively describes and reproduces the formation, stabilization, and decay of humoral immunity (Schwarz et al., 2017; Bagheri-Jamebozorgi et al., 2014), explicitly accounting for physical rates, turnover times, and control mechanisms in adaptive immune system states. It provides a comprehensive analytical tool that allows addressing the complexity of biological systems through symbolic representations grounded in biological knowledge and biophysical principles, which can be mapped into tractable mathematical models and computational simulators. Starting from an ODE model, we construct a phase space that represents a fingerprint of complexity in the AISR, namely the threshold-like immune response to increasing antigen load (Akondy et al., 2015; Tay et al., 2014; Zinkernagel and Hengartner, 2001). This result further demonstrates the power of the ESL modeling tools in capturing emergent behaviors and understanding complex biological systems. The model also suggests that this threshold behavior is controlled by an internal mechanism of mass balance in the bone marrow, which determines microenvironmental interactions in the hematopoietic niche.

Beyond reproducing known physiological behaviors, a key strength of the ESL framework lies in its potential to explore alternative system configurations through scenario-based simulations. Different stock-flow architectures with alternative feedback structures, coupled to the variation of structural parameters within biologically plausible bounds, can be tested to identify distinct dynamical regimes corresponding to different functional or pathological conditions. This makes the approach particularly suitable for addressing “what-if” questions. In the case of the adaptive immune response, this capability provides the tools to explore multiple immunological scenarios—ranging from autoimmune conditions to immunosenescence. AISR is formalized in the ESL to establish a baseline upon which disease states can be framed as targeted perturbations of the same stock–flow diagram. In this framework, Multiple Myeloma (MM) represents a pathological reconfiguration of the same plasma-cell and bone-marrow niche dynamics that sustain AISR in healthy systems (Kumar et al., 2017). In the case of MM, a new population of malignant plasma cells would exploit and reconfigure the bone marrow niche, outcompeting normal counterparts and overriding regulatory constraints (Conte et al., 2023). The result will be the uncontrolled plasma-cell expansion, sustained by altered interactions with space and stromal cells and disrupted feedback loops that normally maintain immune memory. In this context, the AISR model is seen as a healthy baseline contrasting with a disease model (Surra et al., 2025).

### Limitations and future directions

The scope of the present work is limited in several respects, which we state explicitly:

- The structural coefficients are derived from literature on both human and murine studies, as reported in Table 1. Turnover rates of plasma cells and lymphocytes differ between the two species, and the resulting parameter set should be understood as describing a generic mammalian humoral response rather than a species-specific one. Murine data were necessary for specific cellular fluxes (such as bone marrow homing efficiency J9 and stromal niche interaction rates *J*_*5*_), which cannot be non-invasively measured in humans, whereas human clinical data provided parameters for immunoglobulin decay (*J*_*6D*_) and peripheral cell lifespan. While combining cross-species data introduces quantitative scaling uncertainties—since absolute kinetic rates differ between rodents and humans—the ESL framework focuses on structural feedback networks and relative balance of turnover times rather than fitting single-species empirical curves. Sensitivity analyses (Supplementary Figure S1) confirm that the core systemic dynamics, such as memory persistence and threshold response, are topologically robust to parameter variations within biologically plausible cross-species ranges.
- Coefficients that are not derivable from literature or direct measurement are constrained within biophysically plausible bounds, as described in the section on structural parameters. The corresponding uncertainties are assessed by a local, One-Parameter-at-a-Time (OPT) analysis, which measures local derivatives around the baseline configuration. Because the ESL interaction terms are multiplicative, this approach does not capture the global interdependence among parameters. A variance-based Global Sensitivity Analysis is left to future work.
- Using an ATP-equivalent proxy unit allows for a unified accounting of the energetic inventory embodied within distinct system stocks. For cellular and protein stocks the conversion is a physical quantity and corresponds to the biosynthetic cost of the stock as explained in the energetic conversions section. In this regard, the niche-space stock *Q*_*7*_ serves as a capacity-accounting device where one unit of space is defined as the volume released by one long-lived plasma cell. This modeling choice makes the spatial constraint commensurable with the cell stocks and dimensionally consistent within the flow terms, but represents an occupancy capacity rather than actual free energy stored in the niche of a complex tissue environment such as the bone marrow. Future work will build on this foundation to provide a possibly complete biophysical description of the role of space in cell-population based models.
- Our calculations indicate that the total energy embodied in the circulating antibody stock *Q*_*6*_ is substantially larger than that embedded within the long-lived plasma cell stock *Q*_*5*_ responsible for its production. Rather than implying where biological memory functionally resides—since long-lived plasma cells remain biologically essential for sustained antibody secretion—this finding demonstrates that, under our accounting scheme, the energetic inventory stored in circulating immunoglobulins far exceeds the energetic inventory of the cellular machinery maintaining them. Furthermore, assessing the dynamic metabolic cost of humoral immunity requires evaluating the continuous energy flows Ji and power requirements necessary to sustain continuous secretion, rather than analyzing static stock magnitudes in isolation. While the current model establishes a healthy baseline dynamic of adaptive humoral immunity, exploring complex clinical scenarios—such as patient-specific therapeutic responses or malignant reconfigurations like Multiple Myeloma or autoimmune diseases—represents an important direction for future research. By calibrating parameter ranges to individual clinical profiles, future extensions of this framework could potentially contribute to the development of patient-specific ‘disease digital twins’. Similarly, to capture true physiological ageing (immunosenescence), explicit age-dependent decline in microenvironmental capacity *Q*_*7*_ or cellular parameters must be incorporated in subsequent model iterations.
- The AISR model reproduces the ordering, the persistence and the relative magnitude of the regimes of humoral immunity, and its stationary immunoglobulin pool falls within the physiological range. However, quantitative comparison with data from patient cohorts remains a necessary step for understanding the quantitative predictive capabilities of the approach.

## Conclusion

In this paper, we presented a novel approach to the modeling of biomedical systems using Energy Systems Language (ESL). The main points—also illustrated through the application to the dynamics of the immune system response—are summarized as follows:

- The use of ESL to describe and analyze biological systems of medical interest responds to the need to characterize complex behaviors that emerge across multiple spatial and temporal scales. This can be the case of complex diseases whose onset manifests as a systemic malfunction.
- In the case of complex diseases, the analyzed systems are not the biological ones, where the disease takes place and evolves, but rather the disease itself. In this sense, the ESL diagrams do not represent a “photograph” of a physical system, but rather the representation of its dynamics, ruled by the network of interconnections and feedback mechanisms between its elements.
- The ESL language enables a quantitative description of the system through a set of coupled differential equations. In fact, the algebra used in ESL is rooted in the Thermodynamics of open systems, shifting the attention from the biology of the occurrences to their systemic structure.
- The system’s state is defined by an appropriate set of extensive variables (stocks), whose temporal evolution may give access to new knowledge. This is made possible through the adoption of a common energetic unit for describing the flows connecting the stocks and controlling the processes.
- The ESL framework allows for the analysis of system dynamics at multiple time scales, including those not directly accessible through experimental observation. It is also potentially applicable across classes of biomedical systems, as it does not depend on the specific biochemical mechanisms underlying the processes.
- While conceptually abstract, the approach remains firmly grounded in empirical and observational knowledge, particularly in the definition of structural system parameters. Quantitative comparison of system-level model behaviors with clinical data will be an important step for future development of patient-specific computational tools.
- Designed to generate computational simulators capable of answering “what if” scenarios, the methodology opens new possibilities for the systemic characterization of complex biomedical systems. Such simulators can address the creation of suitable “disease digital twins”, for which a general disease can be described by different sets of parameters, depending on the specific patients. The present work is a first step towards this goal and serves as a proof-of-concept framework for related future research.
- A hallmark of system complexity—namely, the emergence of threshold behaviors—can be observed through the extended time-scale simulations enabled by ESL. These behaviors offer valuable insight into the temporal organization and adaptability of living systems.

ESL-based studies in biology and medicine hold significant potential for a wide range of health challenges. In the near future, this framework might be capable of capturing non-linear, emergent dynamics characteristic of conditions such as autoimmune diseases and some forms of hematological cancers (e.g., multiple myeloma). Its ability to simulate disease onset, progression, and variability in therapeutic responses makes it a promising tool for advancing system-level understanding in biomedicine. Further research and development in this field are strongly warranted.

## Data Availability

The datasets presented in this study can be found in online repositories. The names of the repository/repositories and accession number(s) can be found here: https://github.com/federicosurra-venice/AISR_SMAC. The repository includes the scripts required to reproduce the results presented in this work, together with a sensitivity analysis of the model.
