# Supplementary material for: Addressing biomedical complexity using the energy systems language
Source: Front Syst Biol. 2026 Oct 7;6:1903662. doi: 10.3389/fsysb.2026.1903662 (PMC13683192; doi:10.3389/fsysb.2026.1903662)
Supplement: Supplementary file 1 [file DataSheet1.pdf]

Supplementary Materials

Addressing biomedical complexity using the Energy Systems Language

Federico Surra, Francesco Gonella, Luigi Conte, Alessandra Romano

Calculation methods for the structural coefficients

In Energy Systems Language (ESL), the coefficients *k* define the proportionality between stocks and flows. Following Odum's formulation, each coefficient can be obtained by dividing the observed or assumed outflow rate *J* by the corresponding driving stock *Q*:

$$J = k \cdot Q \Rightarrow k = \frac{J}{Q}$$

When there are, for example, two interacting inputs in an interaction symbol, the outflow from the process will depend on both, so:

$$J = k \cdot Q_1 \cdot Q_2 \Rightarrow k = \frac{J}{Q_1 \cdot Q_2}$$

More generally, for *n* interacting stocks:

$$J = k \cdot \Pi(Q_i) \Rightarrow k = \frac{J}{\Pi(Q_i)}$$

In biological applications, such coefficients can also be estimated from the determination of doubling times or half-life values, which represent observable turnover rates of populations or molecules. For processes characterized by an exponential growth (e.g., clonal expansion of *B* cells), the constant *k* is related to the doubling time (T_d) by:

$$k = \frac{ln(2)}{T_d}$$

Conversely, for decay processes (e.g., antibody clearance or plasma-cell death), *k* can be obtained from the half-life ($t_{1/2}$):

$$k = \frac{ln(2)}{t_{\frac{1}{2}}}$$

These relations allow conversion of experimentally measurable timescales into ESL coefficients, ensuring that parameterization is biologically grounded.

Conversion of stocks and rate coefficients into ATP-equivalents

Every state variable of the model is mapped onto the common energetic currency by a linear, stock-specific conversion factor c_i,

$$\tilde{Q}_i = c_i Q_i \qquad \text{(S1)}$$

with $c = 5 \cdot 10^{10}$ ATPeq cell^{-1} for all cellular stocks and for the niche-space stock Q7, and $c = 2.19 \cdot 10^{22}$ ATPeq g^{-1} for the immunoglobulin and antigen stocks. A generic term of the system (Eq. 2) that feeds stock i has the form

$$\frac{dQ_i}{dt} = k \prod_{j \in T} Q_j \qquad \text{(S2)}$$

where T is the set of stocks appearing in that term (a single stock for linear losses, two for the bilinear interaction terms). Substituting $Q_j = \tilde{Q}_j / c_j$ and multiplying both sides by c_i leaves the term in the same functional form, with the transformed coefficient

$$\tilde{k} = k \frac{c_i}{\prod_{j \in T} c_j} \qquad \text{(S3)}$$

Three cases follow directly from Eq. S3: (i) Linear loss terms, in which the stock that is depleted is the stock that appears in the term (e.g. apoptosis and proliferative terms), have $c_i = c_j$ and are therefore numerically invariant. (ii) Bilinear terms in which one of the two stocks is the receiving stock are divided by the conversion factor of the second stock. (iii) Terms transferring across compartments of different nature are multiplied by the ratio of the two factors. Table S1 and S2 reports every coefficient of the model in both native and energetic units.

Table S1. Initial state values of the stocks at simulation time t_0, in native and ATP-equivalent units.

Cell pop.	Reference Stock	Stock	Initial State Value	Ref.	Notes	Conversion factor applied	As converted in ATP-eq
B	Unlimited source, outside the system	Naive B-Cells	10^9 [number of cells]	Lanzavecchia	Total B cells	$\times c_{cell}$	$5.0 \cdot 10^{19}$ ATPeq (5.0 J)
Ag	Limited source, outside the system	Antigens	$2 \cdot 10^6$ [number of antigens]	Mayer	—	$\times c_{Ag}$	$3.6 \cdot 10^9$ ATPeq ($3.6 \cdot 10^{-10}$ J)
T	Limited source, outside the system	T-spec. cells	$8.5 \cdot 10^9$ [number of cells]	Szabolcs	1700 cell/mL in peripheral blood, 3%/6h	$\times c_{cell}$	$4.3 \cdot 10^{20}$ ATPeq (43 J)
D	Limited source, outside the system	D-spec. cells	$4 \cdot 10^7$ [number of cells]	Szabolcs	1700 cell/mL in peripheral blood, 3%/6h	$\times c_{cell}$	$2.0 \cdot 10^{18}$ ATPeq (0.20 J)
T-Ag	$Q_{1,0}$	APC (Antigen-presenting T-cells)	$3.4 \cdot 10^9$ [number of cells]	—	$O(k_1 \cdot T \cdot Ag \cdot dt)$	$\times c_{cell}$	$1.7 \cdot 10^{20}$ ATPeq (17 J)
D-Ag	$Q_{2,0}$	APC (Antigen-presenting cells)	$3.2 \cdot 10^9$ [number of cells]	—	$O(k_2 \cdot D \cdot Ag \cdot dt)$	$\times c_{cell}$	$1.6 \cdot 10^{20}$ ATPeq (16 J)
SPACE	$Q_{7,0}$	Carrying capacity of bone marrow niche	10^9 [number of cells]	—	$Q_{7,MAX}$	$\times c_S$	$1.0 \cdot 10^{20}$ ATPeq (10 J)
—	$Q_{3,0} - Q_{6,0}$	All other stocks	0	—	—	—	0

Conversion factors: $c_{cell} = 5 \cdot 10^{10}$ ATPeq per cell ($\equiv 5 \cdot 10^{-9}$ J; Conte et al., 2023); $c_S = c_{cell}$, since one niche slot is defined as the volume released by one long-lived plasma cell; $c_{Ig} = 2.19 \cdot 10^{22}$ ATPeq per gram of immunoglobulin (4 ATP per peptide bond, mean residue mass 110 g/mol), equivalently $5.3 \cdot 10^3$ ATPeq per IgG molecule; $c_{Ag} = 1.8 \cdot 10^3$ ATPeq per antigen molecule (50 kDa); 1 ATPeq $\equiv 10^{-19}$ J.

Table S2. Stationary state values of the stocks, in native and ATP-equivalent units.

Cell pop.	Reference Stock	Stationary State Value	Ref.	Notes	Conversion factor applied	As converted in ATP-eq
T-Ag	Q_1	$1.25 \cdot 10^9$ [number of cells]	Lanzavecchia	Given that 40% of B becomes Q_3 in 2 min.	$\times c_{cell}$	$6.3 \cdot 10^{19}$ ATPeq (6.3 J)
D-Ag	Q_2	$1.2 \cdot 10^6$ [number of cells]	Szabolcs	CD123+, 8 cells/mL	$\times c_{cell}$	$6.0 \cdot 10^{16}$ ATPeq ($6.0 \cdot 10^{-3}$ J)
B-specific	Q_3	$0.4 \cdot 10^9$ [number of cells]	Pinna	0.4% IgG + B cells	$\times c_{cell}$	$2.0 \cdot 10^{19}$ ATPeq (2.0 J)
H out	Q_4	$0.9 \cdot 10^9$ [number of cells]	Szabolcs	Assumption: (0.01–0.05)% of all peripheral blood leucocytes, given $1.05 \cdot 10^{11}$ bone-marrow cells	$\times c_{cell}$	$4.5 \cdot 10^{19}$ ATPeq (4.5 J)
H in	Q_5	$1.5 \cdot 10^9$ [number of cells]	Szabolcs	Assumption: 1.5–2% of all bone-marrow cells (100,000/µL), given that bone marrow is 1.5% of body weight in a 70 kg man (1 kg ≡ 1 L)	$\times c_{cell}$	$7.5 \cdot 10^{19}$ ATPeq (7.5 J)
Ig B	Q_6	3–5 g	Kenneth	Estimated from the stationary value of Ig in a normal person	$\times c_{Ig}$	$(6.6–11) \cdot 10^{22}$ ATPeq ($6.6 \cdot 10^3$J)
SPACE	Q_7	10^9 [number of cells]	MAX Q_7	—	$\times c_S$	$1.0 \cdot 10^{20}$ ATPeq (10 J)

Conversion factors: $c_{cell} = 5 \cdot 10^{10}$ ATPeq per cell ($\equiv 5 \cdot 10^{-9}$ J; Conte et al., 2023); $c_S = c_{cell}$, since one niche slot is defined as the volume released by one long-lived plasma cell; $c_{Ig} = 2.19 \cdot 10^{22}$ ATPeq per gram of immunoglobulin (4 ATP per peptide bond, mean residue mass 110 g/mol), equivalently $5.3 \cdot 10^3$ ATPeq per IgG molecule; $c_{Ag} = 1.8 \cdot 10^3$ ATPeq per antigen molecule (50 kDa); 1 ATPeq $\equiv 10^{-19}$ J.

Table S3. Biological justification, aggregated processes, supporting evidence, and explicit exclusions for the ESL AISR model (Figure 4).

Stock / Flow	Biological Entity Represented	Aggregated Processes	Supporting Literature Evidence	Deliberately Excluded Mechanisms
Q_1	Helper T-cell cognate help signal	Antigen processing, MHC-II presentation, TCR binding, CD40/CD40L & cytokine signaling	Lanzavecchia et al. (1988); Abbas et al. (2015)	Fine-grained T-cell receptor affinity distributions, Treg suppression dynamics.
Q_2	Dendritic cell antigen presentation	DC antigen capture, maturation, migration to lymph nodes, naive cell priming	Szabolcs et al. (2003); Matsue et al. (1999)	Sub-lineage DC subset specialization (e.g., pDC vs cDC1/cDC2 dynamics).
Q_3	Activated & Memory B cells	Clonal expansion, affinity selection, memory B-cell differentiation	Macallan et al. (2005); Jones et al. (2015)	Explicit somatic hypermutation sequences, class-switch recombination pathways.
Q_4	Short-lived plasma cells & Plasmablasts	Early differentiation, rapid proliferation, initial systemic antibody release	Cassese et al. (2003); Brynjolfsson et al. (2018)	Micro-anatomical position changes within lymph node extrafollicular foci.
Q_5	Long-lived plasma cells (LLPCs)	Bone marrow niche homing, long-term survival program, continuous antibody secretion	Amanna & Slifka (2010); Aaron & Fooksman (2022)	Competition with non-plasma hematopoietic stem cells for stromal niches.
Q_7	Bone marrow niche spatial capacity	Stromal cell docking site availability, CXCL12/APRIL survival factor expression	Aaron & Fooksman (2022); Conte et al. (2023)	Dynamic structural remodeling of bone trabeculae over human aging.

Figure S1

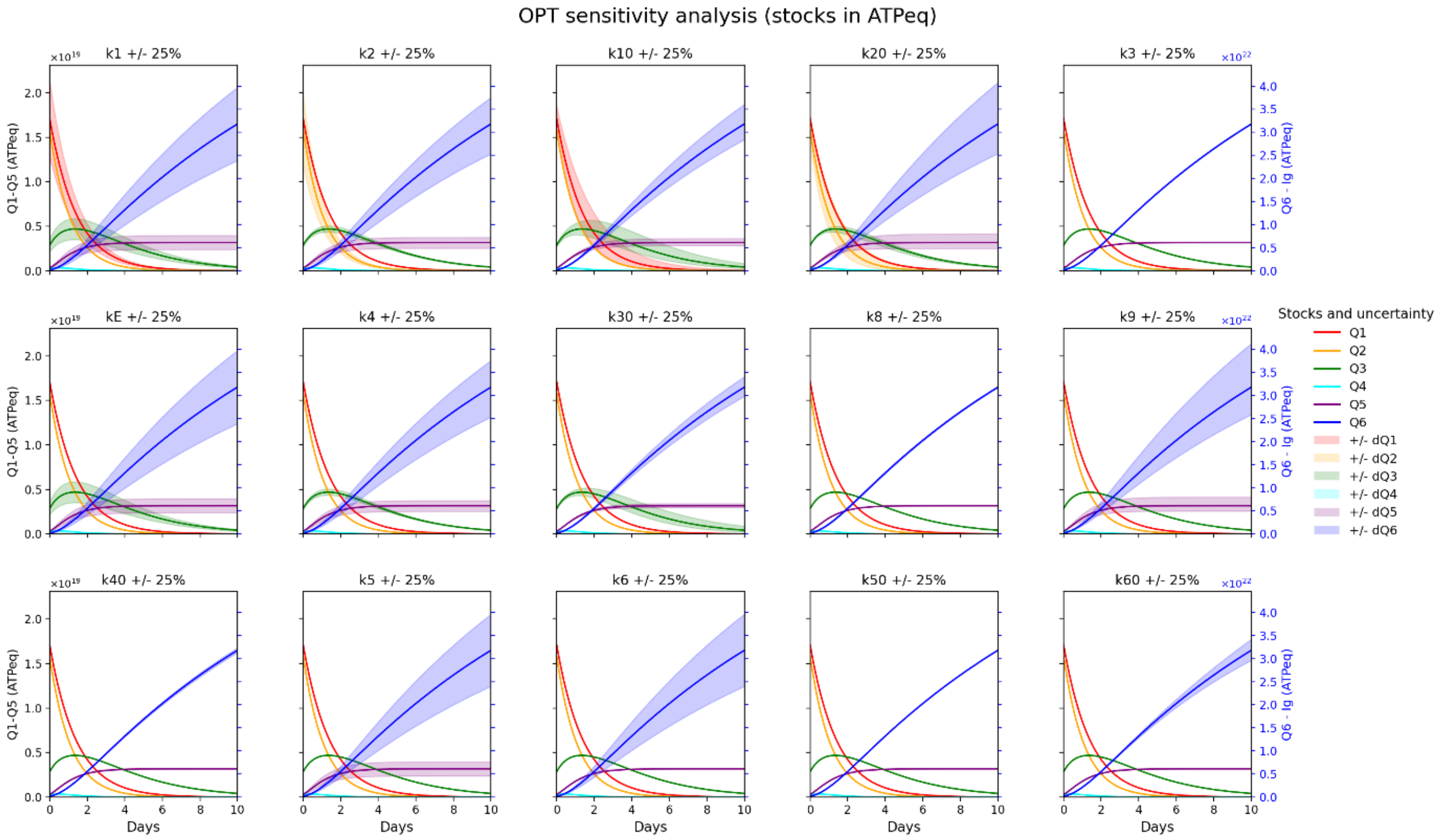
OPT sensitivity analysis (stocks in ATPeq)
k1 +/- 25%
k2 +/- 25%
k10 +/- 25%
k20 +/- 25%
k3 +/- 25%
kE +/- 25%
k4 +/- 25%
k30 +/- 25%
k8 +/- 25%
k9 +/- 25%
k40 +/- 25%
k5 +/- 25%
k6 +/- 25%
k50 +/- 25%
k60 +/- 25%
Q1-Q5 (ATPeq)
Q6 - Ig (ATPeq)
Days
Stocks and uncertainty
Q1
Q2
Q3
Q4
Q5
Q6
+/- dQ1
+/- dQ2
+/- dQ3
+/- dQ4
+/- dQ5
+/- dQ6

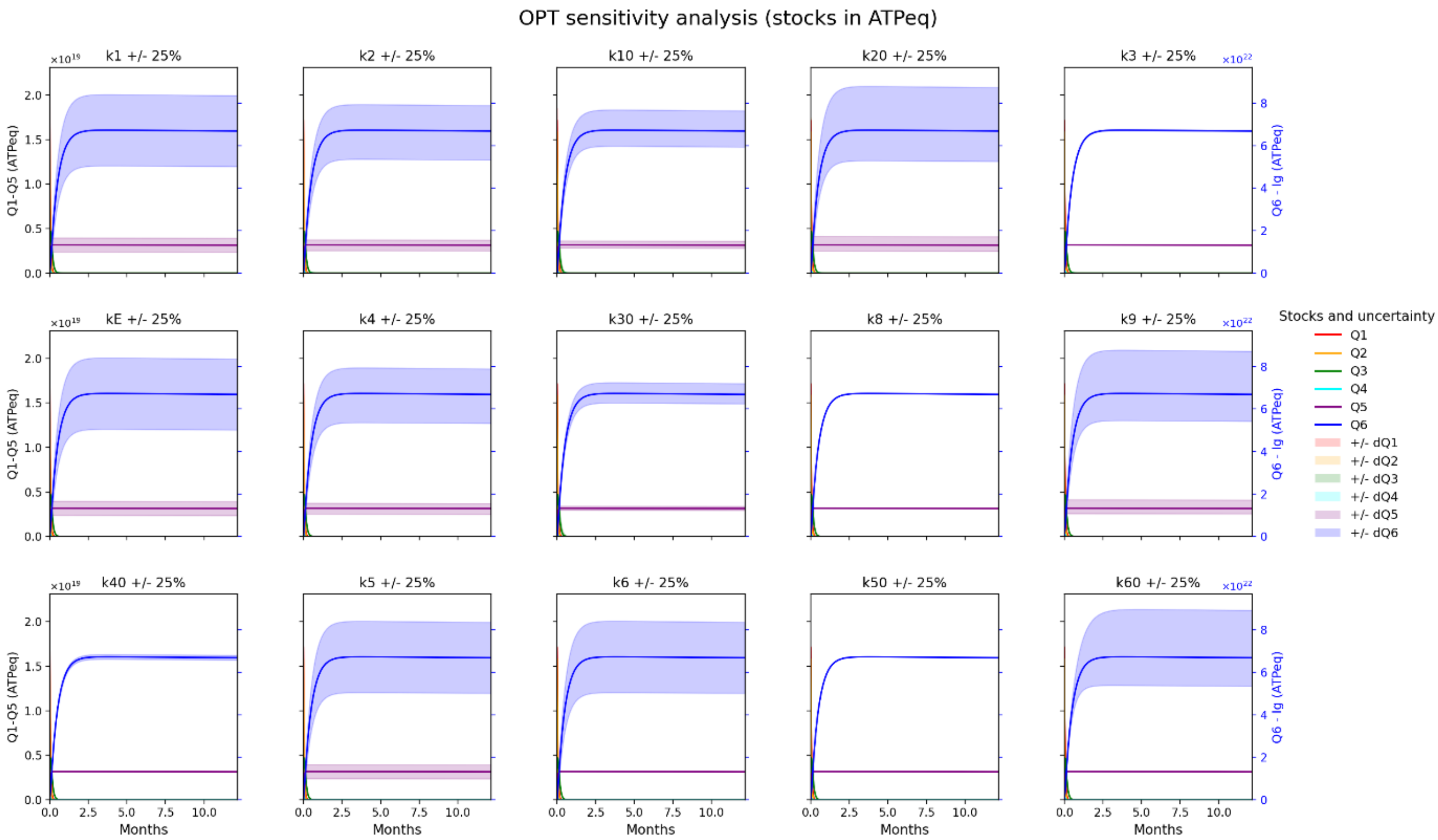
OPT sensitivity analysis (stocks in ATPeq)
k1 +/- 25%
k2 +/- 25%
k10 +/- 25%
k20 +/- 25%
k3 +/- 25%
kE +/- 25%
k4 +/- 25%
k30 +/- 25%
k8 +/- 25%
k9 +/- 25%
k40 +/- 25%
k5 +/- 25%
k6 +/- 25%
k50 +/- 25%
k60 +/- 25%
Q1-Q5 (ATPeq)
Q6 - Ig (ATPeq)
Months
Stocks and uncertainty
Q1
Q2
Q3
Q4
Q5
Q6
+/- dQ1
+/- dQ2
+/- dQ3
+/- dQ4
+/- dQ5
+/- dQ6

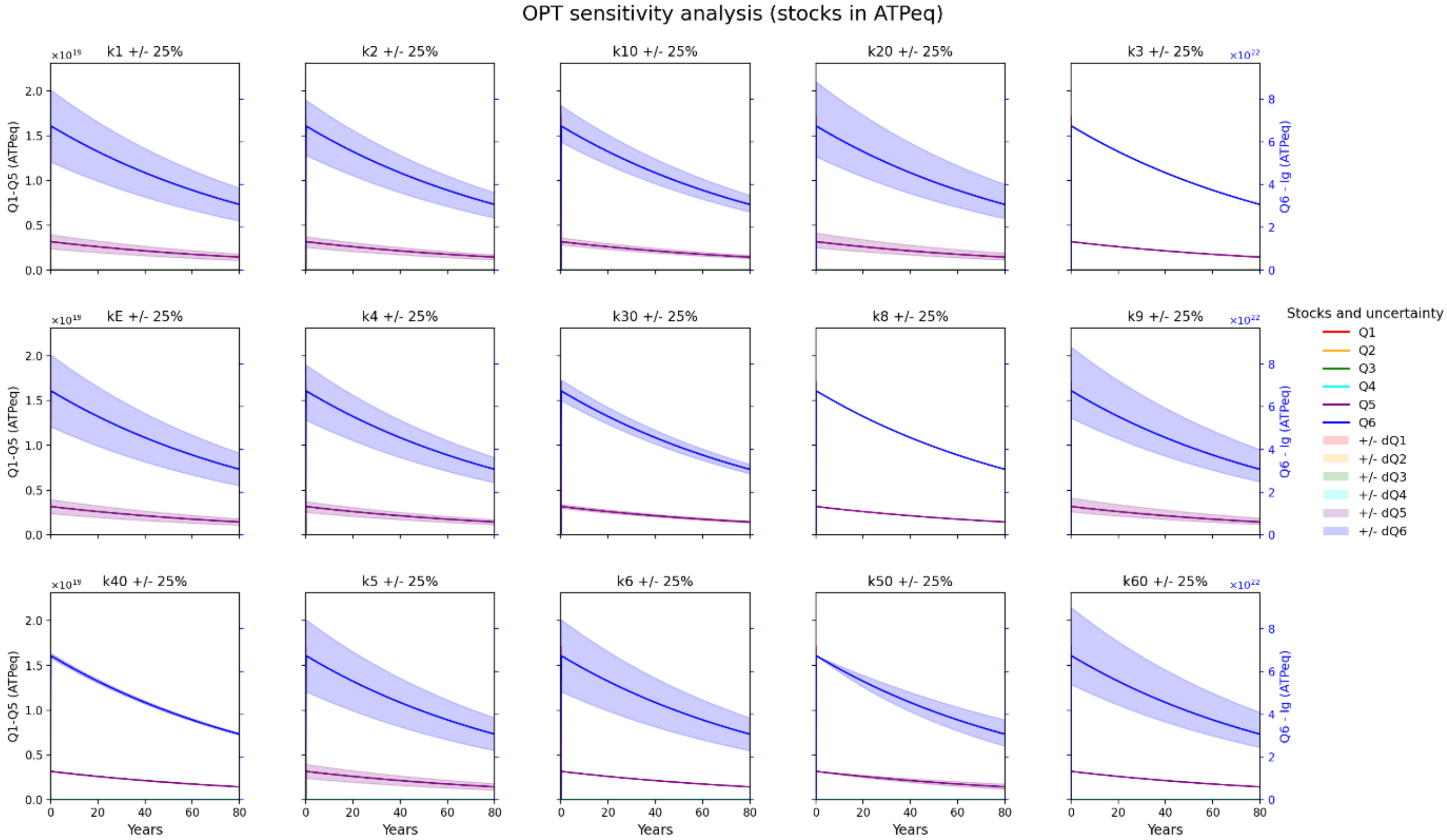

One-parameter-at-a-time (OPT) sensitivity analysis (±25 %) for all rate constants in the adaptive immune response model, shown over three-time horizons. Simulated trajectories are plotted for stocks Q_1 (T-activated antigen precursors, red), Q_2 (D-activated antigen precursors, orange), Q_3 (memory B cells, green), Q_4 (short-lived plasmablasts, cyan), Q_5 (long-lived plasma cells, purple), and Q_6 (circulating immunoglobulin, blue). In each subplot, the solid line represents the baseline trajectory, and the shaded envelope indicates the ±25 % perturbation of a single parameter (Δk_n). The figure shows the stability of the systemic dynamics under structural parameter uncertainty, confirming the robustness of the model settings.

(TOP FIGURE) 0–10-day dynamics: Early *T*- and *B*-cell responses as Q_1 and Q_2 peak immediately and decay within the first few days, followed by transient $Q_3 \rightarrow Q_4$ expansion and the emerging $Q_5 \rightarrow Q_6$ response. Sensitivity bands illustrate which parameters most influence each stock's rise and fall in the acute phase.

(MIDDLE FIGURE) 0–12-month (one-year) response: After Q_1–Q_4 have largely returned to zero, Q_5 (purple) plateaus around month 1 and then decays slowly, while Q_6 (blue) peaks by month 3–4 before its gradual decline. Only parameters affecting plasma-cell survival and antibody clearance produce appreciable shading in this window.

(BOTTOM FIGURE) 0–80-year evolution: Long-term bone-marrow plasma cell (Q_5) and antibody (Q_6) titers decline over decades. The sensitivity envelopes in (C) highlight rate constants governing plasma-cell longevity and continuous Ig secretion as the dominant factors shaping the chronic-phase tail.
